# Causality matters in medical imaging

**DOI:** 10.1038/s41467-020-17478-w

**Published:** 2020-07-22

**Authors:** Daniel C. Castro, Ian Walker, Ben Glocker

**Affiliations:** Biomedical Image Analysis Group, Department of Computing, Imperial College London, South Kensington Campus, London, SW7 2AZ UK

**Keywords:** Computational models, Data processing, Machine learning, Predictive medicine, Medical research

## Abstract

Causal reasoning can shed new light on the major challenges in machine learning for medical imaging: scarcity of high-quality annotated data and mismatch between the development dataset and the target environment. A causal perspective on these issues allows decisions about data collection, annotation, preprocessing, and learning strategies to be made and scrutinized more transparently, while providing a detailed categorisation of potential biases and mitigation techniques. Along with worked clinical examples, we highlight the importance of establishing the causal relationship between images and their annotations, and offer step-by-step recommendations for future studies.

## Abstract (web summary)

Scarcity of high-quality annotated data and mismatch between the development dataset and the target environment are two of the main challenges in developing predictive tools from medical imaging. In this Perspective, the authors show how causal reasoning can shed new light on these challenges.

Tremendous progress has been achieved in predictive analytics for medical imaging. With the advent of powerful machine-learning (ML) approaches such as deep learning, staggering improvements in predictive accuracy have been demonstrated for applications such as computer-aided diagnosis^1^ or assisting radiotherapy planning and monitoring of disease progression via automatic contouring of anatomical structures^2^. However, two of the main obstacles for translating these successes to more applications and into wider clinical practice remain: data scarcity, concerning the limited availability of high-quality training data required for building predictive models; and data mismatch, whereby a model trained in a lab environment may fail to generalise to real-world clinical data.

Let us exemplify with a hypothetical scenario how these obstacles may arise in practice and pose real threats to the success of research projects. Suppose a team of academic radiologists is excited about the opportunities artificial intelligence seems to offer for their discipline. In a recent study, the clinical team was able to demonstrate the effectiveness of using human interpretation of magnetic resonance imaging (MRI) for diagnosis of prostate cancer, yielding higher sensitivity and specificity than a conventional diagnostic test, as confirmed via ground-truth labels from histopathology. Motivated by these results, the team decides to approach a ML research lab with the idea of developing a tool for automated, MRI-based diagnosis of prostate cancer. Because reading MRI requires advanced training and experience, they hope such a system may facilitate widespread adoption of MRI as a novel, accurate, and cost-effective tool for early diagnosis, especially in locations with lower availability of the required human expertise.

The clinicians still have access to their previous study data, and are confident this may be used for ML development. Unfortunately, the sample size is small—there are insufficient pairs of images and diagnosis labels to train a state-of-the-art deep learning image classification method. However, the clinicians have access to large amounts of (unlabelled) routine MRI scans. The ML researchers are hopeful they can additionally leverage this data in a so-called semi-supervised learning strategy. After a pilot phase of development, the team is planning to evaluate their method in a large multi-centre study.

What are the chances of success for their project, and how could a causal analysis help them to identify potential issues in advance? Regarding the limited availability of annotated data, here the team may be lucky in successfully exploiting the unlabelled data thanks to the anticausal direction between images and confirmed diagnosis labels (as we will discuss later in more detail). However, major obstacles may arise due to data mismatch between the ML development and clinical validation stage, resulting from specific inclusion criteria (selection bias), varying patient populations (e.g. changes in demographics), and prevalence of disease (e.g. due to environmental factors). Identifying these issues is important for properly designing prospective validation studies. Although researchers are generally aware of the adverse effects of such differences in aspects of the data, they may be unaware that causal reasoning provides tools for laying out any underlying assumptions about the data-generating process in a clear and transparent fashion, such that any issues can be more easily identified beforehand and possibly resolved by employing suitable data collection, annotation and ML strategies.

In this article, we discuss how causal considerations in medical imaging can shed new light on the above challenges—illustrated with cartoon examples in Fig. 1—and help in finding appropriate solutions. In particular, we demonstrate how the causal structure of a task can have profound, and sometimes surprising, consequences on the soundness of the employed ML approach and resulting analysis. We highlight that being aware of causal relationships, and related issues such as dataset shift and selection bias, allows for systematic reasoning about what strategies to prefer or to avoid. Here, the language of causal diagrams provides explicit means to specify assumptions, enabling transparent scrutiny of their plausibility and validity^3^. It is in fact a natural way of defining the relationships between variables of interest, because it reflects the expert’s knowledge of the biological and logistical processes involved in the generation and collection of data, and has been successfully applied for building models for decision-making in healthcare, for example^4,5^. We hope our work can serve as a practical guide and inspire new directions for research in medical imaging.

**Fig. 1:**
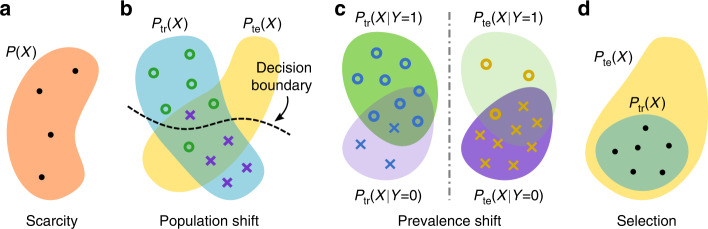
Key challenges in machine learning for medical imaging. **a** Data scarcity and **b**–**d** data mismatch. *X* represents images and *Y*, annotations (e.g. diagnosis labels). *P*_tr_ refers to the distribution of data available for training a predictive model, and *P*_te_ is the test distribution, i.e. data that will be encountered once the model is deployed. Dots represent data points with any label, while circles and crosses indicate images with different labels (e.g. cases vs. controls).

## Causality matters

Before diving into details of the challenges of data scarcity and data mismatch, the causal properties of the core predictive task must be analysed. In particular, one must pay close attention to the relationship between the inputs and targets of the devised model. Readers less familiar with causal reasoning may refer to Box 1 for a brief background and introductory references.

Box 1 Brief background on causal reasoning

Causal reasoning is the process of analysing the data-generating process in terms of cause–effect relationships. One can formalise causation as follows: a variable *A* is a direct cause of variable *B*, written *A* → *B*, if forcing *A* to different values changes the likelihood of *B*, all else held constant^31^. In other words, *B* (say, an outcome) is assumed to have a mechanistic dependence on *A* (say, exposure) and potentially also on other factors and on independent noise^64^. Crucially, *A* → *B* entails that the distribution of the cause, *P*(*A*), does not inform or influence the conditional *P*(*B*∣*A*), a principle known as independence of cause and mechanism^64,67^.

Taking this a step further, the postulated causal links between multiple variables form a directed acyclic graph (DAG), called a causal diagram. Such graphs encode assumptions about direct and indirect causal links and capture probabilistic information about variables such as conditional independences. To illustrate this more concretely, let us analyse some canonical relationships between three variables. If *A* (say, exposure to sunlight) affects a variable *C* (say, skin cancer) indirectly through its impact on *B* (damage to the skin cells), illustrated with the causal diagram in panel a, we say *B* is a mediator and *A* is an indirect cause of *C*. Here, *B* completely screens off the effect of *A* on *C*, meaning *A* ⫫ *C*∣*B* (read ‘*A* is conditionally independent of *C*, given *B*’). Alternatively, assume *A* is a common cause of *B* and *D* (say, vitamin D levels), represented by the causal diagram in panel b. In this case, *A* is known as a confounder, producing an association between *B* and *D*, thus *B* ⊥̸ *D* (read ‘*B* is not independent of *D*’). However, controlling for *A* makes them independent: *B* ⫫ *D*∣ *A*. Finally, consider the case wherein *B* is a common effect of *A* and *E* (say, genetic predisposition), illustrated in panel c. Here, *B* is called a collider. Unlike the two situations above, this configuration implies *A* and *E* are independent a priori. On the other hand, conditioning on *B* introduces an association between *A* and *E*, as they may now ‘explain away’ the effect of each other on the observed outcome, *B* (i.e. *A* ⊥̸ *E*∣*B*)^31^.

For more general graph structures, such as the full example diagram in panel d, one should reason in terms of paths (i.e. chains of nodes connected by edges pointing in any direction), as they are the conduits for correlations propagated across the graph. Any path that does not contain a collider is said to be unblocked or open, and implies a potential statistical association between its endpoints. Conversely, a path containing a collider is said to be blocked or closed, and does not carry any indirect causal influence between its endpoints a priori^65^. If there are no unblocked paths between two variables, we conclude they are independent. As mentioned above, however, conditioning on a collider (or on a descendant of one) may unblock previously blocked paths.

A purely statistical perspective would be unable to distinguish all three configurations in panels a–c, making it difficult to decide what to control for. The causal perspective, on the other hand, requires us to be clear about our assumptions and immediately reveals possible confounding. Under this model, for example, although vitamin D levels are predictive of skin cell damage, taking vitamin supplements would be assumed to have no effect on the sun-damaged DNA molecules. The fact that causal models allow us to enquire about the effects of interventions is what sets them apart from pure statistical models, which are limited to studying correlations. This illustrates that careful considerations may be required when making decisions about the data collection, sample selection, and subsequent analysis. With the ability to formalise causal concepts in clear mathematical and probabilistic terms, causal reasoning opens the door for researchers to go beyond association by allowing them to incorporate domain expertise when answering fundamental scientific questions. We refer the reader to ‘Methods’ section for a more detailed treatment of causality theory, including advice on using domain knowledge to build their own causal graphs.

### Predictive analytics in medical imaging

The focus of this article is on predictive modelling: given an image *X*, train a model to predict some given annotation *Y*. Specifically, we wish to estimate the conditional probability distribution *P*(*Y*∣*X*) by fitting a statistical model with a suitable objective function. This formulation encompasses a variety of common medical image analysis tasks, such as semantic segmentation (i.e. contouring of structures of interest), disease classification, outcome prediction, and many more.

In this context, it is worth clarifying some terminology regarding the data that is used for development and after deployment, in order to avoid confusion of some terms that are sometimes used differently in clinical and ML communities. We refer to an annotated dataset with pairs (*X*, *Y*) as the development data, which is used to train and test a predictive model in a lab environment. In ML workflows, the development data is typically split into training, validation and hold-out test sets. The training set is used to learn the model parameters (e.g. the weights in a convolutional neural network), whereas the validation set is used during training to monitor the learning progress and avoid overfitting to the training set. The test set is used only after training is completed, in order to quantify the performance of the model on ‘unseen’ data. It is prudent to avoid re-using the test data in development cycles as it can lead to unrealistic performance estimates^6^.

Importantly, the assumption that the performance of a trained model on the development test set is representative of the performance on new clinical data after deployment in varying environments is often violated due to differences in data characteristics, as discussed earlier. It is therefore absolutely critical to be able to clearly formalise and communicate the underlying assumptions regarding the data-generating processes in the lab and real-world environments, which in turn can help anticipate and mitigate failure modes of the predictive system.

### Causality in medical imaging

Given the specification of the input images, *X*, and the prediction targets, *Y*, it is imperative to determine which is the cause and which is the effect. Using the categorisation in ref. ^7^, we wish to establish whether a task is

- Causal: estimate *P*(*Y*∣*X*), when *X* → *Y* (predict effect from cause); or
- Anticausal: estimate *P*(*Y*∣*X*), when *Y* → *X* (predict cause from effect).

The answer is crucial to all further causal analysis of the problem, and has a strong impact on the applicability of semi-supervised learning^8,9^ (discussed later) and on whether generative or discriminative models should be preferred^10^.

Recall the definitions of cause and effect: if the annotation could have been different by digitally editing the image beforehand, then one can conclude that the image causes the annotation. For example, manual segmentation masks are drawn over the image by visual inspection and would evidently be influenced by certain pixel changes. On the other hand, a pathology lab result would be unaffected by such manipulations. Images and targets may alternatively be confounded, i.e. descend from a common cause. This relationship is often treated similarly to the anticausal case^7^.

**Fig. 2:**
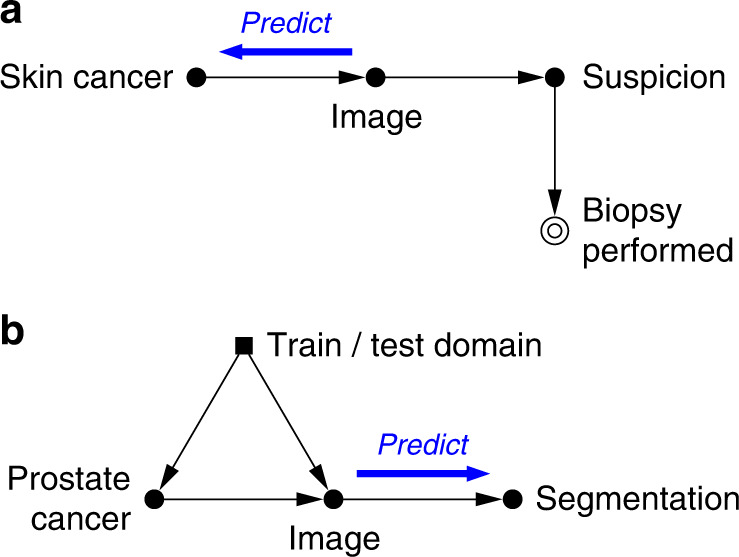
Causal diagrams for medical imaging examples. **a** Skin lesion classification. **b** Prostate tumour segmentation. Filled circular nodes represent measured variables, double circular nodes denote sample selection indicators, and squares are used for sample domain indicators. Here we additionally highlight the direction of the predictive task.

It is generally possible to discern causal structures only when we are aware of the acquired data’s background details, as meta-information plays a fundamental role in understanding the data generation and collection processes. A recently compiled ontology of medical imaging meta-information^11^ contains several attributes that can help characterise the predictive causal direction in an imaging study, such as field of application and task category (e.g. lesion detection for screening, segmentation for treatment planning), as well as details about the annotation process (manual vs. (semi-)automatic vs. laboratory; image-wide vs. pixel-wise annotations; factors affecting reliability; etc.). Let us further illustrate this discussion with two practical examples, depicted in Fig. 2.

### Worked clinical examples

Consider a skin lesion classification task, wherein a set of dermoscopic images (*X*) is collected along with histopathology diagnosis for melanoma following biopsy (*Y*). Here, *Y* is a gold-standard proxy for the true presence of skin cancer, and as such can be considered as a cause of the visual appearance of the lesion, *X*. This task is therefore anticausal (note the arrow directions in Fig. 2a). Further, routine dermoscopic examination of pigmented skin lesions typically results in a ‘benign’, ‘suspicious’, or ‘malignant’ label. Prediction of such labels would instead be causal, as they are obtained visually and could be affected if the images were digitally manipulated.

Now recall our earlier example where a team of radiologists had developed a new MRI-based diagnostic tool for prostate cancer. This time the team aims to improve the cancer treatment via radiotherapy by automating the planning process. Currently, the patient MRI scans (*X*) need to be manually segmented by carefully contouring the tumour regions and any organs-at-risk (*Y*). This annotation is done by visual inspection and evidently depends on image content, resolution, and contrast, for example, whereas manually editing the segmentation masks would have no effect on the images. These considerations allow us to conclude that image segmentation is a case of causal prediction (*X* → *Y*; Fig. 2b).

For the two examples above, establishing the causal direction between images and prediction targets seemed reasonably straightforward. This is not always the case, and arguably in many settings identifying whether the relationship is causal or anticausal can be non-trivial, particularly if crucial meta-information is missing. Consider the case when prediction targets are extracted from radiology reports. At first, one may conclude that the report reflects purely the radiologist’s reading of a medical image, hence image causes report. However, their conclusions could be based on additional information—potentially even more important than the findings in the images—such as blood tests or other diagnostic test results. In the context of segmentation, an annotator’s knowledge about the grade of a tumour might influence how certain boundaries will be contoured, in which case an additional arrow from ‘prostate cancer’ to ‘segmentation’ could be included. This would however not alter the fact that the segmentations are a consequence of the images (and diagnoses), thus the task remains causal. Or what if image-derived diagnosis labels determined by an expert with long years of experience are nearly identical to biopsy results? Could these labels serve as proxies for the ground truth, configuring an anticausal relationship? These instances highlight the importance of investigating and modelling the full data-generating process to make informed decisions about the causal relationships underlying the data. As there may not always be a single correct answer, it is crucial to clearly communicate the assumptions we make so these can be open to scrutiny.

## Data scarcity

One of the notorious challenges in medical image analysis is the scarcity of labelled data, in great part due to the high costs of acquiring expert annotations or expensive lab tests, e.g. to confirm initial diagnosis. The techniques often used to circumvent this shortage, namely semi-supervised learning and data augmentation, have markedly different properties under the lens of causality.

### Tackling data scarcity via semi-supervision

Semi-supervised learning (SSL) aims to leverage readily available unlabelled data in the hope of producing a better predictive model than is possible using only the scarce annotated data. Given this ambitious goal, it is perhaps unsurprising that strong requirements need to be met. Namely, the distribution of inputs needs to carry relevant information about the prediction task—otherwise it would be pointless to collect additional unlabelled data. This idea is typically articulated in terms of specific assumptions about the data which can be intuitively summarised as follows^8^: similar inputs (images in our case) are likely to have similar labels and will naturally group into clusters with high density in the input feature space. Lower density regions in that space in-between clusters are assumed to be ideal candidates for fitting decision boundaries of predictive models. In this context, considering large amounts of unlabelled data together with the scarce labelled data may reveal such low density regions and may lead to better decision boundaries than using labelled data alone.

Note how this idea insinuates an interplay between the distribution of inputs, *P*(*X*), and the label conditional, *P*(*Y*∣*X*). Now recall that, by independence of cause and mechanism, if the prediction task is causal (*X* → *Y*), then *P*(*X*) is uninformative with respect to *P*(*Y*∣*X*), and SSL is theoretically futile in this case^8,9^. Since typical semantic segmentation tasks are causal, as illustrated in our prostate cancer example, there is likely very little hope that semantic segmentation can fundamentally benefit from unlabelled data, which may relate to recent concerns raised in the literature^12^. Intuitively, a model trained on image-derived annotations will attempt to replicate the (most often manual) annotation process, rather than to predict some pre-imaging ground truth (e.g. ‘true’ anatomy). It is plausible, then, that seeing more raw images without corresponding anatomical information provides no new insight about the annotation mechanism. Conversely, if *Y* → *X* as for skin lesions, then these distributions may be dependent, and semi-supervision has a chance of success^9^. We conjecture that, in practice, anticausal problems are more likely than causal ones to comply with the SSL assumptions outlined above, as observed, e.g. among the datasets analysed in ref. ^10^.

That is not to say that SSL is completely useless for causal tasks, as there can be practical algorithmic benefits. Under certain conditions, unlabelled data can be shown to have a regularising effect, potentially boosting the accuracy of an imperfect model by lowering its variance^13^, and may reduce the amount of labelled data required to achieve a given performance level^14,15^. To the best of our knowledge, there have been no empirical studies systematically investigating the efficacy of SSL in causal and anticausal medical imaging tasks, especially for segmentation, hence further work is needed to validate its gains.

A recent comprehensive empirical study^12^ reported that properly tuned purely supervised models and models pre-trained on related labelled datasets (i.e. transfer learning) are often competitive with or outperform their semi-supervised counterparts. It also demonstrated that SSL can hurt classification performance under target shift (discussed later as prevalence shift) between labelled and unlabelled sets. This suggests that practitioners willing to apply SSL should be cautious of potential target distribution mismatch between labelled and unlabelled sets—e.g. unequal proportions of cases and controls or presence of different pathologies.

### Tackling data scarcity via data augmentation

In contrast with SSL, data augmentation produces additional plausible (*x*, *y*) pairs by systematically applying random, controlled perturbations to the data. Because it provides more information about the joint distribution, *P*(*X*, *Y*) rather than only the marginal *P*(*X*), it is suitable for both causal and anticausal tasks, without the theoretical impediments of semi-supervised learning for causal prediction. This now ubiquitous technique is a powerful way of injecting domain knowledge to improve model robustness to variations one expects to find in the test environment. Notably, we can distinguish between augmentations encouraging invariance and equivariance.

Many tasks require predictions to be insensitive to certain types of variation. Examples include image intensity augmentations, such as histogram manipulations or addition of noise, and spatial augmentations (e.g. affine or elastic transformations) for image-level tasks (e.g. regression or classification, as in the skin lesion example). As these augmentations apply uniformly to all inputs *x* without changing the targets *y*, their benefits stem from a refined understanding of the conditional *P*(*X*∣*Y*), while contributing no new information about *P*(*Y*).

For other tasks, such as segmentation or localisation, predictions must change similarly to the inputs, e.g. a spatial transformation applied to an image *x*—such as mirroring, affine or elastic deformations—should be likewise applied to the target *y* (e.g. spatial coordinates or segmentation masks, as in the prostate tumour example). Information is gained about the joint distribution via its shared spatial structure, related to e.g. anatomy and acquisition conditions.

## Data mismatch

The recurrent issue of mismatch between data distributions, typically between training and test sets or development and deployment environments, tends to hurt the generalisability of learned models. In the generic case when no assumptions can be made about the nature of these differences, any form of learning from the training set is arguably pointless, as the test-time performance can be arbitrarily poor. Nonetheless, causal reasoning enables us to recognise special situations in which direct generalisation is possible, and to devise principled strategies to mitigate estimation biases. In particular, two distinct mechanisms of distributional mismatch can be identified: dataset shift and sample selection bias. Learning about their differences is helpful for diagnosing when such situations arise in practice.

### Data mismatch due to dataset shift

Dataset shift is any situation in which the training and test data distributions disagree due to exogenous factors, e.g. dissimilar cohorts or inconsistent acquisition processes. As before, let *X* be the input images and *Y* be the prediction targets. We use an indicator variable *D* for whether we are considering the training (*P*_tr_(*X*, *Y*)) or the test domain (*P*_te_(*X*, *Y*)):

1 $${P}_{{\rm{tr}}}(X,Y):={P}_{D = 0}(X,Y)\quad {\rm{and}}\quad {P}_{{\rm{te}}}(X,Y):={P}_{D = 1}(X,Y).$$

For simplicity, in the following exposition we will refer only to disparities between training and test domains. This definition can however extend to differences between the development datasets (training and test data) and the target population (after deployment), when the latter is not well represented by the variability in the test data.

Moreover, when analysing dataset shift, it is helpful to conceptualise an additional variable *Z*, representing the unobserved physical reality of the subject’s anatomy. We then interpret the acquired images *X* as imperfect and potentially domain-dependent measurements of *Z*, i.e. *Z* → *X*.

**Fig. 3:**
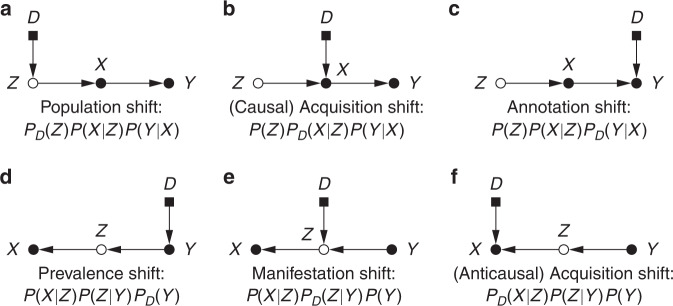
Selection diagrams for dataset shift. **a**–**c** Causal and **d**–**f** anticausal scenarios, with corresponding factorisations of the joint distribution *P*_*D*_(*X*, *Y*, *Z*). *X* is the acquired image; *Y*, the prediction target; *Z*, the unobserved true anatomy; and *D*, the domain indicator (0: ‘train’, 1: ‘test’). An unfilled node means the variable is unmeasured.

Switching between domains may produce variations in the conditional relationships between *X*, *Y* and *Z* or in some of their marginal distributions. Based on the predictive causal direction and on which factors of the joint distribution change or are invariant across domains, dataset shift can be classified into a variety of familiar configurations. Here we formulate the concepts of ‘population shift’, ‘annotation shift’, ‘prevalence shift’, ‘manifestation shift’ and ‘acquisition shift’. These terms correspond roughly to particular dataset shift scenarios studied in general ML literature, namely ‘covariate shift’, ‘concept shift’, ‘target shift’, ‘conditional shift’ and ‘domain shift’, respectively^16^. However, we believe it is beneficial to propose specific nomenclature that is more vividly suggestive of the phenomena encountered in medical imaging. By also explicitly accounting for the unobserved anatomy, the proposed characterisation is more specific and enables distinguishing cases that would otherwise be conflated, such as population or manifestation shift versus acquisition shift. The basic structures are summarised in Fig. 3 in the form of selection diagrams (causal diagrams augmented with domain indicators)^3^, and some examples are listed in Table 1. We hope this may empower researchers in our field to more clearly communicate dataset shift issues and to more easily assess the applicability of various solutions.

**Table 1:** Types of dataset shift.

Type	Direction	Change	Examples of differences
Population shift	Causal	*P*_*D*_(*Z*)	Ages, sexes, diets, habits, ethnicities, genetics
Annotation shift	Causal	*P*_*D*_(*Y*∣*X*)	Annotation policy, annotator experience
Prevalence shift	Anticausal	*P*_*D*_(*Y*)	Baseline prevalence, case–control balance, target selection
Manifestation shift	Anticausal	*P*_*D*_(*Z*∣*Y*)	Anatomical manifestation of the target disease or trait
Acquisition shift	Either	*P*_*D*_(*X*∣*Z*)	Scanner, resolution, contrast, modality, protocol

For causal prediction, we name population shift the case wherein only intrinsic characteristics (e.g. demographics) of the populations under study differ, i.e. *P*_tr_(*Z*) ≠ *P*_te_(*Z*). Fortunately, this case is directly transportable, i.e. a predictor estimated in one domain is equally valid in the other^17^. An underfitted model (‘too simple’) may however introduce spurious dependencies, for which importance reweighting with *p*_te_(*x*)/*p*_tr_(*x*) is a common mitigation strategy^18,19^. This approach is not without limitations, however, as it requires access to *P*_te_(*X*) and may rely on further assumptions in order to truly correct for changes in *P*(*Z*). Moreover, learning in this scenario makes sense only if the variability in the training data covers the support of the test distribution^16^—in other words, there are no guarantees about extrapolation performance to modes of variation that are missing from the training environment.

Under prevalence shift (for anticausal tasks), the differences between datasets relate to class balance: *P*_tr_(*Y*) ≠ *P*_te_(*Y*). This can arise for example from different predispositions in the training and test populations, or from variations in environmental factors. If the test class distribution *P*_te_(*Y*) is known a priori (e.g. from an epidemiological study), generative models may reuse the estimated appearance model *P*_tr_(*X*∣*Y*) (= *P*_te_(*X*∣*Y*)) in Bayes’ rule, and, for discriminative models, instances can be weighted by *p*_te_(*y*)/*p*_tr_(*y*) to correct the bias in estimating the training loss. Alternatively, more elaborate solutions based on the marginal *P*_te_(*X*) are possible^18,20^, or the unknown target prevalence *P*_te_(*Y*) may be approximated using the confusion matrix of a trained predictive model^21^.

Cases of annotation shift involve changes in class definitions, i.e. the same datum would tend to be labelled differently in each domain (*P*_tr_(*Y*∣*X*) ≠ *P*_te_(*Y*∣*X*)). For example, it is not implausible that some health centres involved in an international project could be operating slightly distinct annotation policies or grading scales, or employing annotators with varying levels of expertise (e.g. senior radiologists vs. trainees). Without explicit assumptions on the mechanism behind such changes, models trained to predict *P*_tr_(*Y*∣*X*) evidently cannot be expected to perform sensibly in the test environment, and no clear solution can be devised^22^. A tedious and time-consuming calibration of labels or (partial) re-annotation may be required to correct for annotation shift.

Another challenging scenario is that of manifestation shift, under which the way anticausal prediction targets (e.g. disease status) physically manifest in the anatomy changes between domains. In other words, *P*_tr_(*Z*∣*Y*) ≠ *P*_te_(*Z*∣*Y*). As with annotation shift, this cannot be corrected without strong parametric assumptions on the nature of these differences.

We lastly discuss acquisition shift, resulting from the use of different scanners or imaging protocols, which is one of the most notorious and well-studied sources of dataset shift in medical imaging^23^. Typical pipelines for alleviating this issue involve spatial alignment (normally via rigid registration and resampling to a common resolution) and intensity normalisation. In addition, the increasingly active research area of domain adaptation investigates data harmonisation by means of more complex transformations, such as extracting domain-invariant representations^24,25^ or translating between imaging modalities^26^ (e.g. synthesising MRI volumes from CT scans^27^). Note that domain adaptation may fail or even be detrimental under changes in class prevalence^28^.

Returning to the prostate cancer example, suppose our dataset was collected and annotated for research purposes, employing a high-resolution 3 T MRI scanner and containing a majority of younger patients, and that the trained predictive model is to be deployed for clinical use with conventional 1.5 T scanners. This is a clear case of dataset shift, firstly because the images are expected to be of different quality (acquisition shift). Secondly, because the different age distribution in the target population entails variations in prostate size and appearance (population shift). In addition, the presence of both types of shift can lead to confounding (Fig. 2b): a model trained on this data may erroneously learn that image quality is predictive of the risk of prostate cancer.

### Data mismatch due to sample selection bias

A fundamentally different process that also results in systematic data mismatch is sample selection. It is defined as the scenario wherein the training and test cohorts come from the same population, though each training sample is measured (*S* = 1) or rejected (*S* = 0) according to some selection process *S* that may be subject-dependent:

2 $${P}_{{\rm{tr}}}(X,Y):=P(X,Y| S=1)\quad {\rm{and}}\quad {P}_{{\rm{te}}}(X,Y):=P(X,Y).$$

**Fig. 4:**
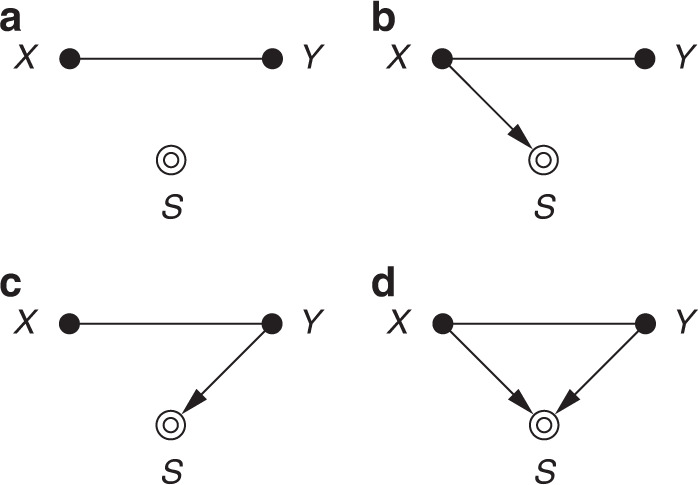
Causal diagrams for different sample selection scenarios. **a** Random; **b** image-dependent; **c** target-dependent; **d** jointly dependent. *S* = 1 indicates an observed sample, and plain edges represent either direction.

Some examples are presented in Table 2. The main difference to standard dataset shift is the data-dependent selection mechanism (Fig. 4), as opposed to external causes of distributional changes (Fig. 3). In other words, the indicator variables in sample selection concern alterations in the data-gathering process rather than in the data-generating process^19^.

**Table 2:** Types of sample selection.

Type	Causation	Examples of selection processes	Resulting bias
Random	None	Uniform random sampling	None
Image	*X* → *S*	Visual phenotype selection (e.g. anatomical traits, lesions)	Population shift
		Image quality control (QC; e.g. noise, low contrast, artefacts)	Acquisition shift
Target	*Y* → *S*	Hospital admission, filtering by disease, annotation QC, learning strategies (e.g. class balancing, patch selection)	Prevalence shift
Joint	*X* → *S* ← *Y*	Combination of the above (e.g. curated benchmark dataset)	Selection bias^30^

Completely random selection simply corresponds to uniform subsampling, i.e. when the training data can be assumed to faithfully represent the target population (*P*_tr_(*X*, *Y*) ≡ *P*_te_(*X*, *Y*)). Since the analysis will incur no bias, the selection variable *S* can safely be ignored. We conjecture this will rarely be the case in practice, as preferential data collection is generally unavoidable without explicit safeguards and careful experimental design.

Selection can be affected by the appearance of each image in two different manners. We can select subjects based on anatomical features—viewing the image *X* as a proxy for the anatomy *Z*—which has similar implications to population shift. Alternatively, selection criteria may relate to image quality (e.g. excluding scans with noise, poor contrast, or artefacts), which is akin to acquisition shift^22^. If selection is purely image-based (*X* → *S*, cf. Fig. 4b), we may exploit the conditional independence *S* ⫫ *Y*∣*X*, which implies that the predictive relation is directly recoverable^29^, i.e. *P*_te_(*Y*∣*X*) ≡ *P*_tr_(*Y*∣*X*). In a learning scenario, however, the objective function would still be biased, and methods for mitigating the corresponding cases of dataset shift can be employed. Relating back to the skin lesion example, patients are referred for biopsy only if dermoscopy raises suspicions. As inclusion in this study is image-dependent, a dataset with ground-truth biopsy labels is not representative of the overall distribution of pigmented skin lesions.

When selection is solely target-dependent (*Y* → *S*), we have *P*_te_(*X*∣*Y*) ≡ *P*_tr_(*X*∣*Y*), and it can be treated as prevalence shift. This will typically result from factors like hospital admission, recruitment or selection criteria in clinical trials, or annotation quality control. Notably, ML practitioners should be wary that it can also arise as a side-effect of certain training strategies, such as class re-balancing or image patch selection for segmentation (e.g. picking only patches containing lesion pixels).

Sample selection can additionally introduce spurious associations when the selection variable *S* is a common effect of *X* and *Y* (or of causes of *X* and *Y*): implicitly conditioning on *S* unblocks an undesired causal path between *X* and *Y* (see Methods). This is the classic situation called selection bias^30^ (cf. Berkson’s paradox^31^), and recovery is more difficult without assumptions on the exact selection mechanism. In general, it requires controlling for additional variables to eliminate the indirect influence of *X* on *Y* via conditioning on the collider *S*^3,29^.

## Discussion

This paper provides a fresh perspective on key challenges in machine learning for medical imaging using the powerful framework of causal reasoning. Not only do our causal considerations shed new light on the vital issues of data scarcity and data mismatch in a unifying approach, but the presented analysis can hopefully serve as a guide to develop new solutions. Perhaps surprisingly, causal theory also suggests that the common task of semantic segmentation may not fundamentally benefit from unannotated images via semi-supervision. This possibly controversial conclusion may prompt empirical research into validating the feasibility and practical limitations of this approach.

Other advanced topics could be worth exploring in future work for causally expressing more subtle facets of predictive modelling workflows. In particular, one recurring topic in epidemiology and sociology that is relevant to our imaging context is measurement bias^32,33^. This is the study of properties of proxy variables, which stand in for true variables of interest that are difficult or impossible to measure directly. Of particular note are the cases wherein proxies are additionally affected by other variables (‘differential’), or when measurement errors for separate proxies are correlated (‘dependent’)^34^. Measurement bias was explored here for the case of acquisition shift (images as proxies for anatomy, affected by the domain), and similar considerations could extend to other variables, e.g. patient records or pathology results.

A further pertinent topic is that of missingness. Whereas sample selection refers to the observability of full records, missingness concerns partial measurements—i.e. when some subjects may be missing observations of some variables. This is the context of semi-supervised learning, for example, as target labels are observed only for a subset of the data points. The classical characterisation distinguishes whether data is missing completely at random, missing at random, or missing not at random, when the missingness of a measurement is independent of any of the variables of interest, dependent on observed variables, or dependent on the missing values, respectively^35^. Causal diagrams again prove instrumental in identifying such structural assumptions about missingness mechanisms^36,37^.

Finally, we highlight that our contribution is only the first step towards incorporating causality in medical image analysis. Here we introduce to this community purely the language of causal reasoning, hoping this will facilitate novel research directions exploiting causality theory to its full extent. Specifically, the endeavours of causal inference and causal discovery are so far largely unexplored in medical imaging. In this context, they could lead to the discovery of new imaging biomarkers and to exciting new applications such as personalised counterfactual predictions (‘What if a patient were not a smoker?’). Large population imaging studies such as the UK Biobank^38,39^ can greatly empower this kind of research, as they offer unique opportunities for extracting the relevant patterns of variation from sheer observational data.

Beside enabling new research directions, incorporation of causal reasoning in medical image analysis aligns with a growing awareness among stakeholders of the need for responsible reporting in this field. There have been increasing efforts from regulatory bodies—such as the US Food and Drug Administration^40,41^, the UK’s Department of Health and Social Care^42^, National Institute for Health and Care Excellence^43^, and NHSX^44^, and even the World Health Organization^45^—to outline best practices for the safe development and monitoring of AI-enabled medical technologies^46^. Guidelines for designing and reporting traditional clinical trials are now also being specialised for AI-based interventions^47^. This has been accompanied by a recent surge in discussion among the medical community about the opportunities and, crucially, the risks of deploying such tools in clinical practice^48–55^. Most of the apprehension revolves around the external validity of these predictive models, i.e. their generalisability beyond the development environment, in terms of e.g. robustness to dataset shift^48,49^ and selection bias^48,56^, as discussed herein. Other important concerns involve data inaccuracy, inconsistency, and availability^48–50,53,56^, and alignment of the model training objective with the target clinical setting^49,51,53,54^. In a similar yet complementary vein to the notable TRIPOD guidelines^57,58^, our work ties precisely into this context of encouraging transparent reporting of predictive analytics in healthcare.

This debate also relates to parallel initiatives from within the machine learning community, in specific in the emerging field of fairness, accountability, and transparency (FAT). Scholars in FAT have proposed checklist-style guidelines for reporting datasets^59^ and models^60^, for example, and have been investigating sources of failure for ML models, among which is poor reporting^61^. Interestingly, the same formalism of causal reasoning explored here was also shown to be especially well-suited for expressing and addressing issues of unfairness (e.g. social biases)^62^ and dataset shift^63^ in other contexts.

Overall, the goal of this article has been to introduce to the medical imaging community the language of causal diagrams, and to demonstrate how it can illuminate common issues in predictive modelling. While causal reasoning by itself may not solve any of the data scarcity or mismatch problems, it provides a clear and precise framework for expressing assumptions about the data. Presenting such assumptions transparently in the form of causal diagrams makes them immediately recognisable by other researchers, and therefore easier to be confirmed or disputed. The real challenge lies in identifying these very assumptions, as they can often be unclear or ambiguous.

**Fig. 5:**
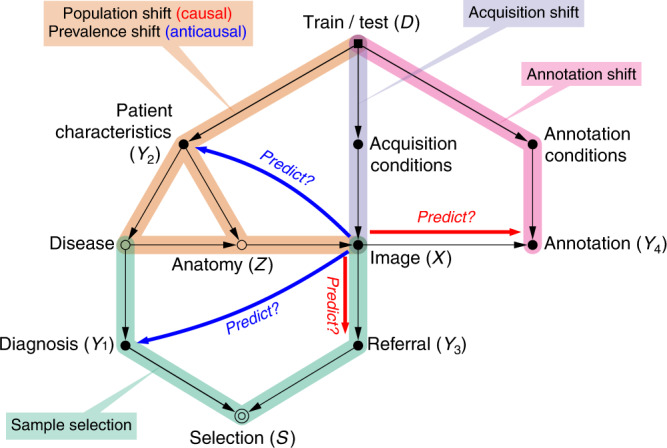
A ‘scaffold’ causal diagram summarising typical medical imaging workflows. We believe most practical cases can be adapted from this generic structure by removing or adding elements. Here are represented a variety of possible prediction targets (marked *Y*_1_–*Y*_4_): some anticausal (*Y*_1_, *Y*_2_) and others, causal (*Y*_3_, *Y*_4_). ‘Annotation’ here refers to any image-derived data, such as lesion descriptions, regions of interest, spatial landmark coordinates, or segmentation maps. Note that annotators will often be aware of the patients' records and diagnoses, in which cases there could be additional arrows from *Y*_1_ or *Y*_2_ towards *Y*_4_.

To facilitate this task, we offer in Table 3 a step-by-step summary of our recommendations, and Fig. 5 presents a generic ‘scaffold’ diagram from which most typical workflows can be adapted. Readers may then refer to the other tables for help in identifying the components of their own diagram for the problem at hand. We believe that this exercise of building the full causal story of a dataset will encourage analysts to consider potential underlying biases more thoroughly, and that it may, like the TRIPOD checklist, lead to ‘more comprehensive understanding, conduct, and analysis of prediction model studies’^58^.

**Table 3:** Step-by-step recommendations.

1. Gather meta-information about the data collection and annotation processes to reconstruct the full story of the dataset
2. Establish the predictive causal direction: does the image cause the prediction target or vice versa? If annotations are scarce and image → target, semi-supervised learning may be futile, while data augmentation remains a viable alternative
3. Identify any evidence of mismatch between datasets (Table 1). When applicable, importance reweighting is a common mitigation strategy; see further specific advice in the text
• If causal (image → target): population shift, annotation shift
• If anticausal (target → image): prevalence shift, manifestation shift
4. Verify what types of differences in image acquisition are expected, if any. Consider applying data harmonisation techniques and domain adaptation (if test images are available)
5. Determine whether the data collection was biased with respect to the population of interest, and whether selection was based on the images, the targets or both (Table 2). Refer to dataset shift guidance for mitigating the resulting biases
6. Draw the full causal diagram including postulated direction, shifts and selections

## Methods

### Fundamentals of causal reasoning

Learning tasks can be broadly divided into three categories based on the causal information used: (i) prediction, in which observed data are used to infer values of unobserved variables, e.g. image classification; (ii) interventions, where investigators study the impact of forcing a variable to attain a certain value, e.g. randomised controlled trials (RCTs) for drug testing; and (iii) counterfactual analysis, wherein observed data combined with a structural causal model are used to answer questions of the form, ‘What would have happened if individual *I* had received treatment *T* instead?’ While most are familiar with causal inference in the context of RCTs or scientific experiments, causal information is vital even in certain purely predictive tasks, as we discussed in the context of medical imaging.

Let us now illustrate the concept of causation and the principle of independence of cause and mechanism, presented earlier in the text. Consider the example wherein a radiologist makes a decision, *B*, for referral to further clinical testing (e.g. needle biopsy) based on any suspicious findings in the patient’s medical scan, *A*. Given an image, the distribution over possible decisions is the conditional *P*(*B*∣*A*). If the appearance of the scan changes, this referral distribution—reflecting the radiologist’s judgement—changes as well. On the other hand, the mechanism that translates from a finding of a suspicious pattern in the scan *A* to a referral decision *B* is independent of how likely any individual scan is to appear in the real world, *P*(*A*). This is further justified as such mechanism may typically be formed by rules from radiology guidelines. Here, the cause of the referral decision is clearly the medical scan, as altering the decision would not affect the scan’s appearance.

In the above example, the correct graphical model would be *A* → *B*, as resolved via domain knowledge. If presented only with observational data of medical images and referrals, however, from a purely statistical perspective one would find it difficult to identify whether *A* → *B* or *B* → *A*. It may still be possible to identify which is the correct relationship if the gathered data were the result of two experiments, respectively manipulating *A* or *B*. Determining the presence and direction of causal relationships from data is the realm of causal discovery, which is an extremely challenging and active field of research but is beyond the scope of this article.

### Causal graphical models

When multiple variables are involved, causal assumptions can be expressed as a simple directed acyclic graph (DAG; no loops, at most one edge between any pair of nodes), whose nodes represent variables of interest and edges between them indicate postulated direct causal influences. Such a causal graphical model, referred to as a causal diagram, embodies the causal Markov assumption (or local Markov): every node is statistically independent of its non-effects (non-descendants), given its direct causes (parents). Therefore, the joint probability distribution over all variables *V*_*i*_ on the graph can be factorised as a product of independent conditional mechanisms^64,65^:

3 $$P({V}_{1},{V}_{2},\ldots ,{V}_{N})=\mathop{\prod }\limits_{i = 1}^{N}P({V}_{i}| {\rm{pa}}({V}_{i})),$$

where pa(*V*_*i*_) denotes the set of parents of variable *V*_*i*_, i.e. the nodes with arrows pointing toward *V*_*i*_.

For those familiar with Bayesian networks, it appears that there is nothing new. However, Bayesian networks only encode conditional independence relationships, and they are thus not unique for a given observational distribution^31^. In fact, although causal arguments often guide the construction of such models, any alignment between arrows in Bayesian networks and causality is merely coincidental. In particular, causal models differ from Bayesian networks in that, beside representing a valid factorisation of the joint probability distribution, they enable reasoning about interventions^31^. In causal graphs, the values for each node are assumed to be determined via independent mechanisms (cf. independence of cause and mechanism) given their direct causes. An intervention is defined as any forced change to the value or distribution of a node, regardless of its direct causes, and results in a modified graph wherein this node is disconnected from its parents, though crucially all other mechanisms are unaffected. This can also be thought of as replacing the mechanism generating a variable by a function independent of its former direct causes (e.g. a constant). Incidentally, this is the principle behind randomised controlled trials: a treatment is assigned at random (an intervention on the ‘treatment’ variable), isolating its direct effect on the outcome by eliminating the influence of confounding factors (i.e. cutting the edges from common causes of treatment and outcome). Note that considering interventions on image and referral decision is also what allowed us to determine the causal direction in the example above.

### Building a causal diagram

The first step in constructing a causal model for a given system is to elicit the relevant variables to represent, which may be observed or not. These ought to be well defined: they should unambiguously correspond to real or postulated entities of the system, and separate variables must not have overlapping meanings^66^. In the medical imaging context, variables normally correspond to the collected data elements, such as images, meta-information fields, labels, patient records, etc. Not all important variables need to be concrete and measurable, however. Other relevant abstract concepts can be instantiated if they help in describing complex processes: e.g. ‘annotation policy’, ‘patient’s health status’, ‘proprietary image post-processing pipeline’.

Secondly, the causal links between the defined variables must be determined. While each added arrow between two nodes in the graph corresponds to assuming causation, it is important to consider that the absence of an arrow also encodes a strong assumption. Namely, that there is no direct causal effect—any marginal association between those variables is fully explained via mediator variables or common causes (see below). In addition, the granularity of ‘direct effects’ is only relative to the chosen level of abstraction^65^. One may wish to detail the complete chain of effects between two causally linked variables, or represent them by a single arrow (e.g. *A* → *B*_1_ → *B*_2_ → *C* vs. *A* → *C*).

In what is called a selection diagram^17^, one also includes special indicator variables that identify the ‘domain’ or ‘environment’, e.g. training vs. testing or which hospital in a multi-site study. Their direct causal effects (outgoing arrows) represent the specific mechanisms through which one assumes the observed populations differ, whereas the absence of a link from a domain selector to a variable implies the latter’s mechanism is invariant across environments^17^. Domain indicators should normally be represented by root nodes in the diagram, with no incoming edges, as they embody exogenous changes to the data distributions. A causal diagram may additionally be augmented with selection variables, when the dataset is subject to preferential subsampling from the population (e.g. inclusion criteria for a clinical trial). The incoming arrows to such a node represent the various selection criteria (deliberate or otherwise) that impacted the collection of the dataset of interest.

Finally, note that this construction is an iterative process. Once a full version of the diagram is written, one must verify that the assumptions implied by the graph match the domain knowledge (see following notes on interpretation), and corrections should be made as needed. Further, recall the diagram’s intent as a communication tool when choosing its level of abstraction, as there is often a tradeoff to be made between accuracy and clarity: the graph should be sufficiently detailed not to omit relevant variables and pathways, though no more complex than necessary^66^.

### Interpreting causal diagrams

Causal diagrams offer a clear language to describe and communicate assumptions made about the underlying data-generating processes. Direct and indirect causal links between variables can be read from a diagram by following directed paths, while any missing connections between variables are equally important indicators that no direct relationship is being assumed. Careful interpretation of a diagram gives insights about potential biases that are important to take into account when designing experimental studies and when drawing conclusions from statistical analysis.

In causality, what is usually referred to as bias is any spurious correlation between two variables, contributed by unblocked paths beside the relationship of interest (Box 1). The ‘classic’ prototypical configurations inducing such biases are confounding (unadjusted common cause; cf. Simpson’s paradox^31^) and collider bias (conditioning on a common effect; cf. selection bias, Berkson’s paradox^31^), and are widely studied in statistical literature^30^. This article in specific focused on how dataset shift results from unblocked paths between domain indicators and relevant variables, and on the consequences of (implicitly) conditioning on selection variables. For example, in a multi-site study wherein age distributions vary across sites, it would be useful to include age alongside the image as inputs to the predictive model, to block the ‘site  →  age  →  image’ path causing population shift. This is what is normally meant in the context of predictive modelling, as in statistics and causal inference, by ‘adjusting/controlling for’ or ‘conditioning on’ a variable. Though interpreting causal diagrams may require practice, it is a worthwhile endeavour that may help with the identification and mitigation of potential issues with the predictive model.
